# Enterokinase Enhances Influenza A Virus Infection by Activating Trypsinogen in Human Cell Lines

**DOI:** 10.3389/fcimb.2018.00091

**Published:** 2018-03-23

**Authors:** Hideki Hayashi, Yoshinao Kubo, Mai Izumida, Etsuhisa Takahashi, Hiroshi Kido, Ko Sato, Mutsuo Yamaya, Hidekazu Nishimura, Kou Nakayama, Toshifumi Matsuyama

**Affiliations:** ^1^Medical University Research Administrator, Nagasaki University School of Medicine, Nagasaki, Japan; ^2^Program for Nurturing Global Leaders in Tropical and Emerging Communicable Diseases, Graduate School of Biomedical Sciences, Nagasaki University, Nagasaki, Japan; ^3^Department of Clinical Medicine, Institute of Tropical Medicine, Nagasaki University, Nagasaki, Japan; ^4^Division of Enzyme Chemistry, Institute for Enzyme Research, Tokushima University, Tokushima, Japan; ^5^Virus Research Center, Clinical Research Division, Sendai Medical Center, Sendai, Japan; ^6^Department of Advanced Preventive Medicine for Infectious Disease, Tohoku University Graduate School of Medicine, Sendai, Japan; ^7^Department of Cancer Stem Cell Biology, Graduate School of Biomedical Sciences, Nagasaki University, Nagasaki, Japan

**Keywords:** enterokinase, influenza A virus, hemagglutinin processing, transmembrane serine protease, genome structure and function

## Abstract

Cleavage and activation of hemagglutinin (HA) by trypsin-like proteases in influenza A virus (IAV) are essential prerequisites for its successful infection and spread. In host cells, some transmembrane serine proteases such as TMPRSS2, TMPRSS4 and HAT, along with plasmin in the bloodstream, have been reported to cleave the HA precursor (HA_0_) molecule into its active forms, HA_1_ and HA_2_. Some trypsinogens can also enhance IAV proliferation in some cell types (e.g., rat cardiomyoblasts). However, the precise activation mechanism for this process is unclear, because the expression level of the physiological activator of the trypsinogens, the TMPRSS15 enterokinase, is expected to be very low in such cells, with the exception of duodenal cells. Here, we show that at least two variant enterokinases are expressed in various human cell lines, including A549 lung-derived cells. The exogenous expression of these enterokinases was able to enhance the proliferation of IAV in 293T human kidney cells, but the proliferation was reduced by knocking down the endogenous enterokinase in A549 cells. The enterokinase was able to enhance HA processing in the cells, which activated trypsinogen *in vitro* and in the IAV-infected cells also. Therefore, we conclude that enterokinase plays a role in IAV infection and proliferation by activating trypsinogen to process viral HA in human cell lines.

## Introduction

Influenza viral infections are an impending threat to humans because recent globalization has increased the likelihood of pandemics happening with these highly infectious pathogens (Richard and Fouchier, 2016; Saunders-Hastings and Krewski, 2016). The life cycle of influenza A virus (IAV) begins when it adsorbs to a host cell, it enters it, becomes uncoated, proliferates, assembles into viral particles, and is finally released when it buds away from the cell. The cycle is sophisticatedly regulated by many viral and cellular factors (Medina and García-Sastre, 2011). Environmental factors, including the host immune system, also affect viral proliferation and infectivity (Duan and Thomas, 2016). One such critical factor is a group of trypsin-like proteases. These proteases activate viral hemagglutinin (HA), which is required for viral adsorption to sialic acids on the target-cell surface and in the uncoating process that releases viral genomic RNA into the host cell (Fontana and Steven, 2015; Blijleven et al., 2016). Trypsin-like proteases are located across various sites in the host's body. The transmembrane serine proteases (TMPRSSs), which include TMPRSS2, TMPRSS4, and HAT, are present within cells or on cell surfaces. In contrast, some trypsin-type proteases are secreted in the tissues, and plasminogen occurs in the blood. These proteases activate the precursor HA (designated HA_0_) by cleavage at specific sites to generate the mature HA_1_ and HA_2_ conformations (Goto and Kawaoka, 1998; Choi et al., 2009; Kido, 2015). Various trypsin genes (PRSS1, PRSS2, and PRSS3) are expressed in non-pancreatic cells in humans (Wang et al., 2008; Yamamoto-Tanaka et al., 2014; Ghilardi et al., 2015), and some ectopic trypsins can enhance IAV proliferation in rat cardiomyoblasts and the lungs (Pan et al., 2011; Indalao et al., 2017). However, the activation mechanism used by PRSS trypsinogens is unclear, because expression of the physiological activator enterokinase (EK, or TMPRSS15) is reported to be restricted to duodenal epithelial cells. Because the presence of an EK on duodenal cells activates the trypsinogens that are secreted into the alimentary tract from the pancreas to digest food (Kitamoto et al., 1995; Zheng et al., 2009), we hypothesized that a small but substantial amount of EK is likely expressed in many non-duodenal cells, where it activates trypsinogens to cleave HA_0_ on the influenza viruses, thereby facilitating their infection and proliferation process.

Here, we cloned the cDNAs encoding two EK isoforms (canonical EK and the EK-X2 isoform) from the mRNAs present in human cells, A549 (a lung cell line) and Huh7 (a liver cell line), respectively. A possible role for EK in HA processing during IAV infection was investigated via exogenous expression of EK in 293T cells and the knocking down of endogenous EK in A549 cells.

## Materials and methods

### Cells and IAV infection

The following cells are derived from humans. NB9 neuroblastoma cells were purchased from RIKEN BioResource Center (http://ja.brc.riken.jp/), and maintained in RPMI 1640 medium supplemented with 15% fetal bovine serum. Capan-2 pancreatic adenocarcinoma cells were purchased from the American Type Culture Collection (ATCC, https://www.atcc.org/en.aspx). A549 lung adenocarcinoma cells and H292 lung mucoepidermoid carcinoma cells were kindly provided by Dr. Yuji Ishimatsu, Department of Cardiopulmonary Rehabilitation Sciences, Nagasaki University Graduate School of Biomedical Sciences, Nagasaki, Japan. HT1080 fibrosarcoma cells, HeLa cervical epithelial carcinoma cells, Caco-2 colon adenocarcinoma cells, TE671 rhabdomyosarcoma cells, U937 monocyte-like histiocytic lymphoma cells 293T human embryonic kidney cells, A431 epidermoid carcinoma cells, and Huh7 hepatocellular carcinoma cells were obtained from already-existing collections, and have been maintained in our laboratory for a long time. They were cultured in Dulbecco's modified Eagle's medium (DMEM) supplemented with 10% fetal bovine serum, except for the U937 cells, which were cultured in RPMI 1640 medium supplemented with 10% fetal bovine serum. MDCK canine kidney cells were purchased from Dainippon Pharmaceutical Co., Ltd, Osaka, Japan, and cultured in Eagle's minimal essential medium (E-MEM) supplemented with 10% fetal bovine serum.

We used the influenza A/WSN/1933(H1N1) strain in most experiments, except the experiment shown in **Figure 8**, in which the influenza A/Aichi/2/68 (H3N2) strain was used. Human cells (1 × 10^5^ of each) were plated in a 24-well plate and inoculated with different amounts of the WSN strain of IAV (multiplicity of infection [MOI] = 0.001–0.1, diluted with 0.1% bovine serum albumin [BSA] in medium) at 37°C for the indicated periods. After the removal of the supernatant, the cells were lysed with 100 μl of GLB lysis buffer (Promega Corporation, Madison, WI, USA). The lysates were cleared by centrifugation at 20,000 × g for 2 min at 4°C, combined with a 1/3 volume of 4 × SDS-PAGE sample buffer, separated electrophoretically, and subjected to western blotting. For the experiment shown in **Figure 8**, 293T cells and 293T cells expressing EK-X2 (clone #7-6) in a 24-well plate were inoculated with the influenza A/Aichi/2/68 (H3N2) strain (MOI = 0.1), which was allowed to adsorb for 1 h at 34°C. The inoculum was removed and replaced with DMEM containing 0.1% BSA with or without 5 μg/ml trypsin. After incubation for 48 h at 37°C, the supernatant was collected and analyzed with a plaque assay. Briefly, MDCK cells were inoculated with serial 10-fold dilutions of the samples, overlain with 2% agar containing 5 μg/ml trypsin, and incubated at 37°C for 48 h. The agar was removed and the cells were stained with 0.1% crystal violet.

### Focus forming assays

Focus forming assays were performed according to the method described previously (Matrosovich et al., 2006). In short, MDCK cells (3 × 10^4^ of each) were seeded onto a 96-well plate and inoculated with five-fold serial dilutions of supernatant (collected from infected cells) in E-MEM containing 0.1% BSA at 35°C and allowed to adsorb. After 1 h of incubation, an equal volume of 1.2% Avicel (colloidal cellulose, Sigma-Aldrich #435244,) containing E-MEM, 10% fetal bovine serum, and 90 μg/ml N-tosyl-l-phenylalanyl chloromethyl ketone (TPCK)-treated trypsin (Worthington Biomedical Co.) was added to the wells. The plate was mixed by tapping, and incubated at 37°C for 24 h. The plated cells were fixed with 3.7% formaldehyde and immuno-stained with an anti-IAV nucleoprotein antibody (Sino Biological Inc.) and TrueBlue peroxidase substrate (KPL) to count the foci.

### cDNA cloning and reverse transcription (RT)–PCR

Total mRNAs were extracted from the human cells with the standard acid guanidinium thiocyanate–phenol–chloroform method, and the cDNAs were synthesized with an oligo(dT)_18_ primer and ProtoScript II Reverse Transcriptase (New England Biolabs Inc., Ipswich, MA USA). The primers used to amplify the different genes from the cDNAs are listed in Table 1. For the PCR, the same amounts of total RNAs were used, and amplified in 45 cycles (denaturation for 10 s at 98°C, annealing for 10 s at 60°C, and extension for 30 s at 70°C) with KOD-FX DNA Polymerase (Toyobo Co., Ltd, Osaka Japan) on an Applied Biosystems LightCycler 1.5 thermocycler (Thermo Fisher Scientific Inc., Waltham, MA USA), unless otherwise stated.

**Table 1:** Primer sequences used for RT-PCR.

**Genes**	**Forward**	**Reverse**
**Enterokinase**		
For all variants	GCACCTGATGGCCACTTAAT	CCAGTCACTGCTGACGAGAG
To discriminate among variants^a^	GAGTCATGAAGCCAGAGCGACATTTA	TGTCTTCGTCAGAACCATCTGGACA
**TMPRSS2**^b^	TAACTGGTGTGATGGCGTGT	CCGCTGTCATCCACTATTCC
**TMPRSS4**	CTGAACAGCCTCGATGTCAA	CAAGGGACAGTCCAGCTCTC
**HAT**^c^	TCACCAGCTACACASGAATACAG	GAAATTTCATGACAACATCCGC
**PRSS1**^d^	CCACCCCCAATACGACAGGAA	TAGTCGGCGCCAGAGCTCGC
**PRSS2**^d^	CCACCCCAAATACAACAGCCG	GGGTAGTCGGCACCAGAACTCAG
**PRSS3**^e^	CGCCACCCTAAATACAACAGGGA	TGGGTAGTCAGCACCAAAGCTCAG
β**-Actin**^f^	ACTGGGACGACATGGAGAAA	GGGGTGTTGAAGGTCTCAAA

The cDNAs were amplified in 45 cycles (10 s denaturation at 98°C, 10 s annealing at 60°C, and 30 s extension at 70°C) except for

a *annealing temperature: 66°C*.

b *annealing temperature: 63°C*.

c *annealing temperature: 55°C*.

d *annealing temperature: 62°C*.

e *annealing temperature: 58°C*.

f *amplified in 40 cycles*.

To clone the full-length EK cDNA, we amplified the overlapping N-terminal and C-terminal halves of the cDNA separately, using the cDNAs from A549 cells and Huh7 cells as the templates and the specific primers listed in Table 2, and then combined them at the unique *Ppu*MI restriction site. To introduce a mutation at the conserved triad H–D–S (Choi et al., 2009), we used the PrimeSTAR Mutagenesis Basal Kit (Takara Bio Inc., Kusatsu, Japan). The sequence was changed from TCAGGAGGACCA (Ser^971^GlyGlyPro in canonical EK) to GCAGGAGGGCCC (Ala^971^GlyGlyPro in the EK mutant), introducing an *Apa*I restriction site that did not affect the coding amino acid sequence, to distinguish the mutant from the wild type easily with *Apa*I digestion. To change the wild-type Ser^1001^ of EK-X2 to the mutant Ala^1001^, the wild-type C-terminal half of EK-X2 was replaced with the mutant C-terminal half of EK at the unique *Ppu*MI site. All constructs generated with PCR were confirmed with DNA sequencing. Other cDNAs, encoding TMPRSS2, TMPRSS4, HAT, PRSS1, PRSS2, and PRSS3, were also cloned with RT–PCR from the total RNA from the human cells listed above, using the specific primers listed in Table 2. All the constructs were confirmed with DNA sequencing.

**Table 2:** Primer sequences used for cloning cDNAs.

**Region**	**Forward**	**Reverse**
**Enterokinase**		
N-terminal half^a^	ATGGGGTCGAAAAGAGGCATATCTTCTAGG	CTCAAAAGGTCCTCCACAGTCCGTAGG
C-terminal half	CCTACGGACTGTGGAGGACCTT	GAGTAGAATGGGAAAATAATGCGAC
**TMPRSS2**^b^	CAAGATGGCTTTGAACTCAGGGT	AGGACGAAGACCATGTGGATTAGC
**TMPRSS4**	AGCATGTTACAGGATCCTGACAGTGATCAACCTC	AGCATTACAGCTCAGCCTTCCAGA
**HAT**	AAAATGTATAGGCCAGCACGTG	ACTTGTTGCACTAGATCCCAGTTT
**PRSS1**	CCACCATGAATCCACTCCTGAT	GAGACTGAAGAGATACTGGGGGC
**PRSS2**	CCACCATGAATCTACTTCTGATCCT	GACCAGGGGCTTTAGCTGTTGG
**PRSS3**	CCACCATGAATCCATTCCTGAT	GAGACTGCAGAGGGACCGGG

The cDNAs were amplified in 45 cycles (10 s denaturation at 98°C, 10 s annealing at 60°C, and 2 min extension at 70°C) except for

a *annealing temperature: 65°C*.

b *annealing temperature: 58°C*.

### Establishment of stable cell lines

To establish stable 293T clones expressing EK or EK-X2, we transfected the cells with a pcDNA vector (Thermo Fisher) expressing the EK or EK-X2 cDNA together with a hygromycin-resistance gene. After selection with 150 μg/ml hygromycin B (Thermo Fisher), several independent clones expressing EK or EK-X2 were isolated and analyzed.

To establish stable A549 cells, we used a lentiviral vector (the pLVX series, TaKaRa) to express small hairpin RNAs (shRNAs) under the control of the U6 promoter (the target sequences knocked down are listed in Table 3), together with a puromycin-resistance gene or blasticidin-resistance gene under the control of the PGK promoter. The recombinant lentiviruses were constructed by simultaneously transfecting 293T cells with the pLVX vector and lentiviral plasmids expressing Gag/Pol, Rev, and VSVG (Naldini et al., 1996). After selection with 1.3 μg/ml puromycin (Nacalai Tesque, Inc., Kyoto, Japan), the puromycin-resistant A549 cells expressing RIG-I-targeting shRNA were pooled, and stored for analysis and subsequent experiments. The sh-RIG-I A549 cells were then infected with recombinant lentiviruses expressing shRNAs targeting different sites in the EK mRNA (sh-EK#1, sh-EK#2, and control shRNA), and selected with 180 μg/ml blasticidin S (Wako Pure Chemical Industries, Ltd, Osaka, Japan). The blasticidin-resistant cells were pooled and analyzed.

**Table 3:** Target sequences to knockdown using shRNA.

**Genes**	**Target sequence**
RIG-I	CCAGAATTATCCCAACCGATATCAT
Enterokinase #1	GGATGACATTAGCCTAACATATGG
Enterokinase #2	GGAGTTACATATAATCCTAATTTG

### IAV processing *in vitro*

We obtained IAV-HA_0_-rich lysates from U937 lymphoma cells (2 × 10^5^ cells in a 24-well plate) infected with IAV (MOI = 0.1) for 48 h, because IAV proliferates better in lymphoma cells than in 293T cells without HA_0_ processing for 48 h. To lyse the cells in the 24-well plate, we used 100 μl of GLB lysis buffer (Promega) per well. The IAV-HA_0_-rich lysates were incubated with N-tosyl-l-phenylalanyl chloromethyl ketone (TPCK)-treated trypsin (0, 3, or 9 μg/ml) or lysate including either EK or EK-X2 for 30 min at room temperature. The reaction was terminated by adding a 1/3 volume of 4 × SDS-PAGE sample buffer. The samples were subjected to electrophoresis and western blotting with an anti-HA antibody.

### Trypsin activity assay

Trypsin activity was monitored as the amount of p-nitroaniline (pNA) released from a specific substrate, measured spectrophotometrically at 405 nm (Colorimetric Trypsin Activity Assay Kit, BioVision, Inc., Milpitas, CA, USA; Hayashi et al., 2011). The substrate and 293T cell lysates exogenously expressing trypsinogen (PRSS1, PRSS2, or PRSS3) were incubated with the 293T cell lysates exogenously expressing various transmembrane serine proteases (TMPRSS2, TMPRSS4, HAT, or EKs) at room temperature for the indicated periods. The amount of released pNA from the substrate by trypsinogen (PRSS1, PRSS2, or PRSS3) activated with the indicated transmembrane serine proteases was measured as the trypsinogen-activating function.

### Western blotting

Cell lysates were separated with 10% SDS-PAGE (Bio-Rad Laboratories, Inc. Hercules, CA, USA) and transferred to polyvinylidene difluoride membranes (Merck Millipore Corporation, Darmstadt, Germany). To detect the specific proteins, an anti-HA antibody (Sino Biological Inc., Beijing, China #11684-RP01), anti-EK antibody (Sigma-Aldrich Co. LLC, St. Louis, MO, USA #HPA015611), anti-PRSS3 antibody (ABGENT San Diego, CA, USA #AP11927c), or anti-RIG-I antibody (Ana Spec Inc., Fremont, CA, USA #54285) was used at a dilution of 1:500–1:2,000. The proteins were visualized with horseradish-peroxide-conjugated anti-rabbit IgG antibody (Bio-Rad), and enhanced chemiluminescent reagent (Bio-Rad).

### Statistical analysis

Quantitative data were analyzed using Student's *t-*test with a 2-tailed *p*-value. The *n* for each analysis is represented in the Figure legends. A value of *p* < 0.05 was considered statistically significant.

## Results

### Expression of TMPRSSs and PRSSs

**Figure 1:**
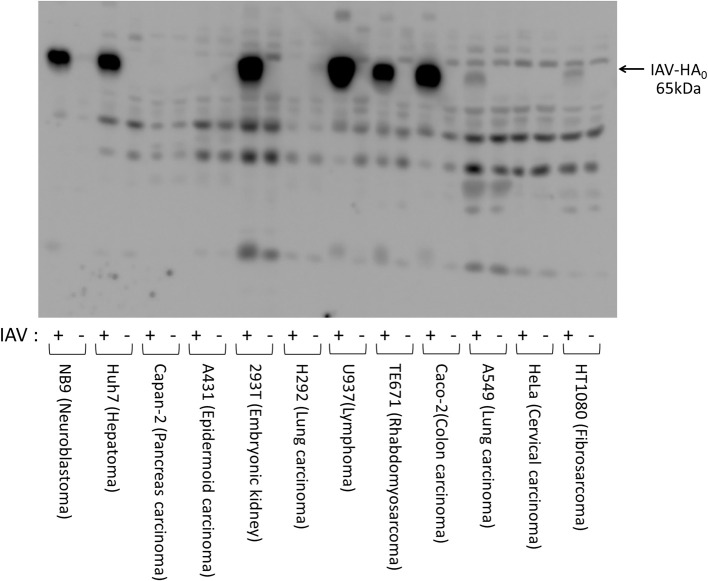
Expressions of HA after infection of IAV [A/WSN/1933(H1N1)] in various human cell lines. Human cell lines (1 × 10^5^ cells) of various origins (HT1080, fibrosarcoma; HeLa, cervical epithelial carcinoma; A549, lung adenocarcinoma; Caco-2, colon adenocarcinoma; TE671, rhabdomyosarcoma; U937, monocyte-like histiocytic lymphoma; H292, lung mucoepidermoid carcinoma; 293T, human embryonic kidney; A431, epidermoid carcinoma; Capan-2, pancreatic adenocarcinoma; Huh7, hepatocellular carcinoma; and NB9, neuroblastoma) were plated in a 24-well plate and inoculated with IAV [A/WSN/1933(H1N1)] (MOI = 0.1). The cell lysates were prepared 48 h after IAV infection, separated electrophoretically, and subjected to western blotting with a specific antibody directed against IAV HA to estimate the amount of viral proliferation in the cells. The 65-kDa precursor IAV HA_0_ protein is indicated with arrow.

**Figure 2:**
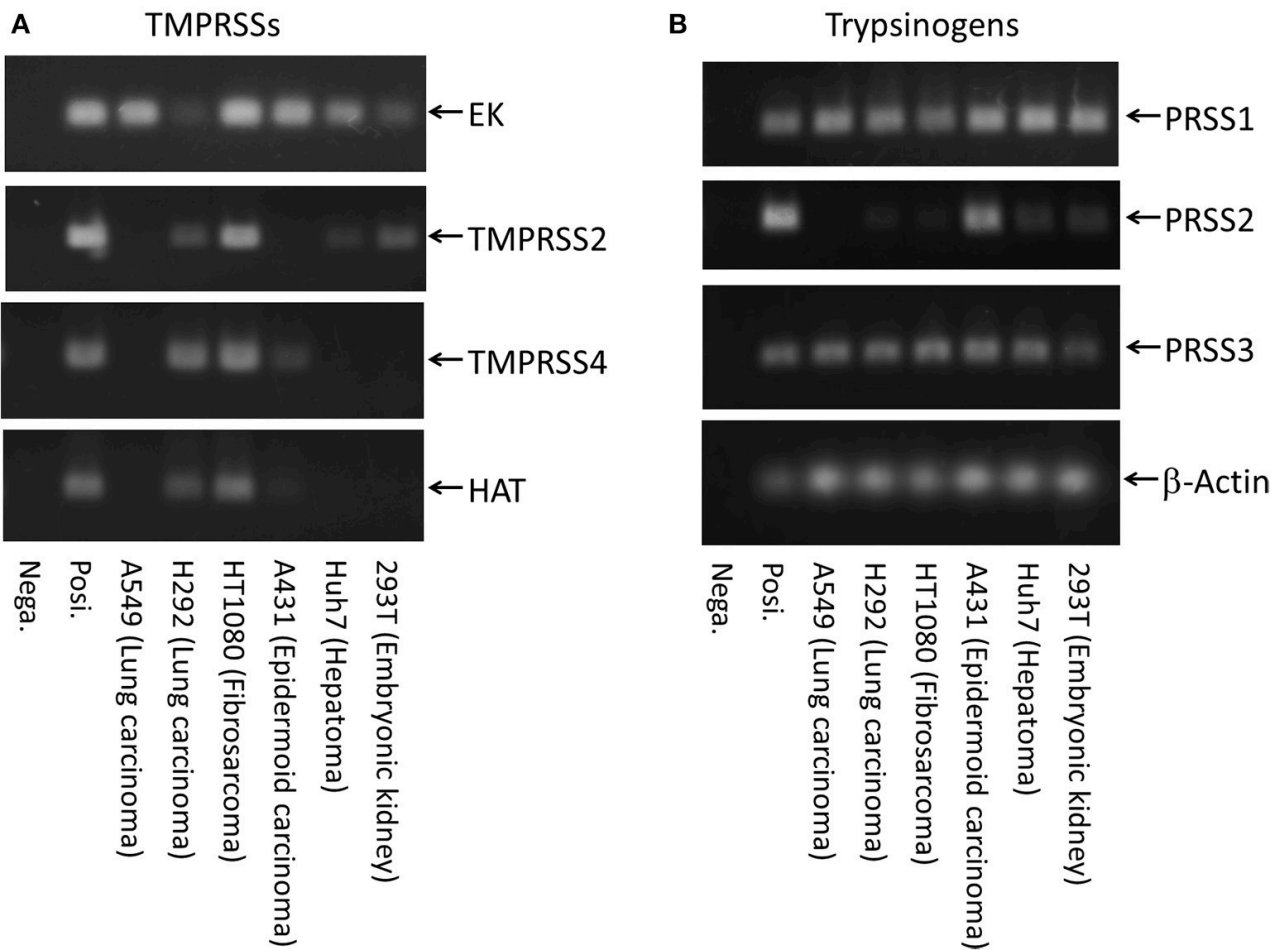
Expression of transmembrane serine proteases and trypsinogens. Total mRNAs from several human cell lines (293T, embryonic kidney; Huh7, hepatoma; A431, epidermoid carcinoma; HT1080, fibrosarcoma; H292, lung carcinoma; and A549, lung carcinoma) shown in Figure 1 were prepared, and their cDNAs were synthesized from the same amounts of total RNA with an oligo(dT)_18_ primer. The transcript copies of the TMPRSS genes **(A)** and trypsinogen genes **(B)** were amplified by 45 cycles of PCR with primers specific for each gene, separated electrophoretically and stained. Posi.: 1 × 10^4^ molecules of target cDNA was used as each positive control. Nega.: H_2_O.

We first examined HA expression 48 h after initiating IAV infections [A/WSN/1933(H1N1)] in various human cell lines (Figure 1). High levels of HA_0_ protein were detected in the IAV-inoculated Caco-2, TE671, U937, 293T, Huh7, and NB9 cells, suggesting that IAV replicates efficiently in these cells. Low levels of HA_0_ protein were detected in HT1080- and A549-inoculated cells, but almost no HA_0_ protein was detected in HeLa, H292, A431, or Capan-2 cells. To evaluate the factors involved in HA processing, we further examined the expression profiles of transmembrane serine proteases (TMPRSSs) and trypsinogens in the cells, using RT–PCR and specific primers (Figure 2). EK was ubiquitously expressed in all the cells we examined, whereas TMPRSS4, TMPRSS2, and HAT were expressed in only some of them (Figure 2A). The lung-derived A549 cells expressed EK, but not TMPRSS4, TMPRSS2, and HAT. In contrast, the other lung-derived H292 and HT1080 fibrosarcoma cells expressed substantial level of TMPRSS4, TMPRSS2, and HAT as well as EK. Considering the HA expression patterns in Figure 1 (e.g., high in 293T and Huh7, low in HT1080 and A549, and almost non-existent in H292 and A431), the TMPRSS expression profile was clearly not directly related to HA expression. As for the trypsinogen genes (PRSS1, PRSS2, and PRSS3 in human cells), PRSS1 and PRSS3 were expressed ubiquitously, but the expression profile of PRSS2 was low in A549, H292, and HT1080 cells (Figure 2B). Taken together, it is clear that each cell line expressed some TMPRSSs and PRSSs that are capable of processing HA_0_ to the active form, although the specific molecules responsible for HA expression and processing were not identified.

**Figure 3:**
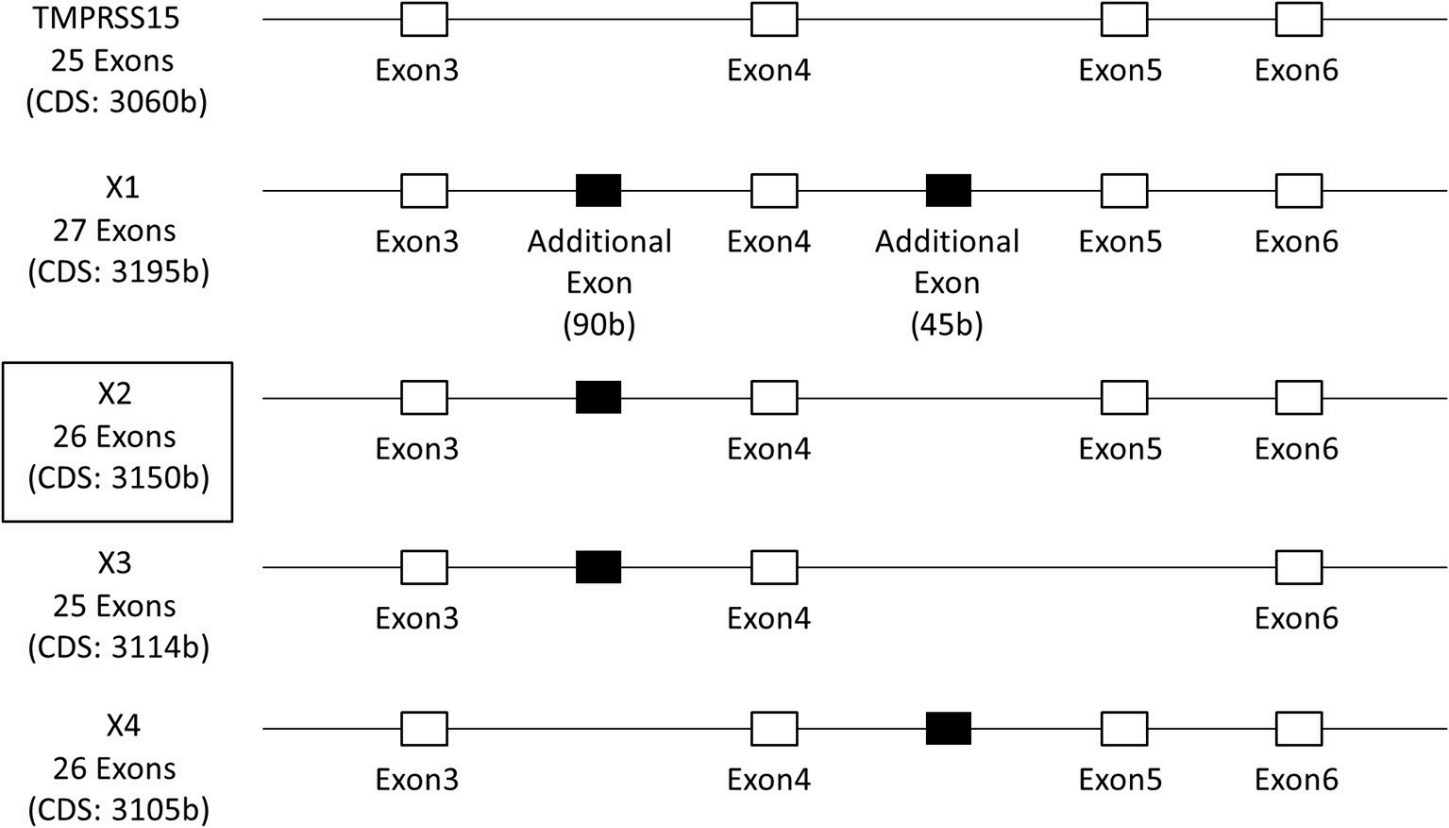
Schematic structure of the EK gene. The 5′-region of EK gene is shown as an open box, containing exons 3–6 of the total 25 exons of canonical EK (TMPRSS15; accession number NM_002772.2), and the additional exons of isoforms X1–X4 (XM_011529654.1–011529659.1), deduced from the GenBank data, are shown as solid black boxes. The three transcript variants (XM_011529654.1–011529656.1) encode the same isoform X1 with different 5′ untranslated regions. The boxed X2 isoform (XM_011529657.1) was actually cloned from Huh7 liver cells, and contains an additional 90-bp exon between exons 3 and 4 of canonical EK.

**Figure 4:**
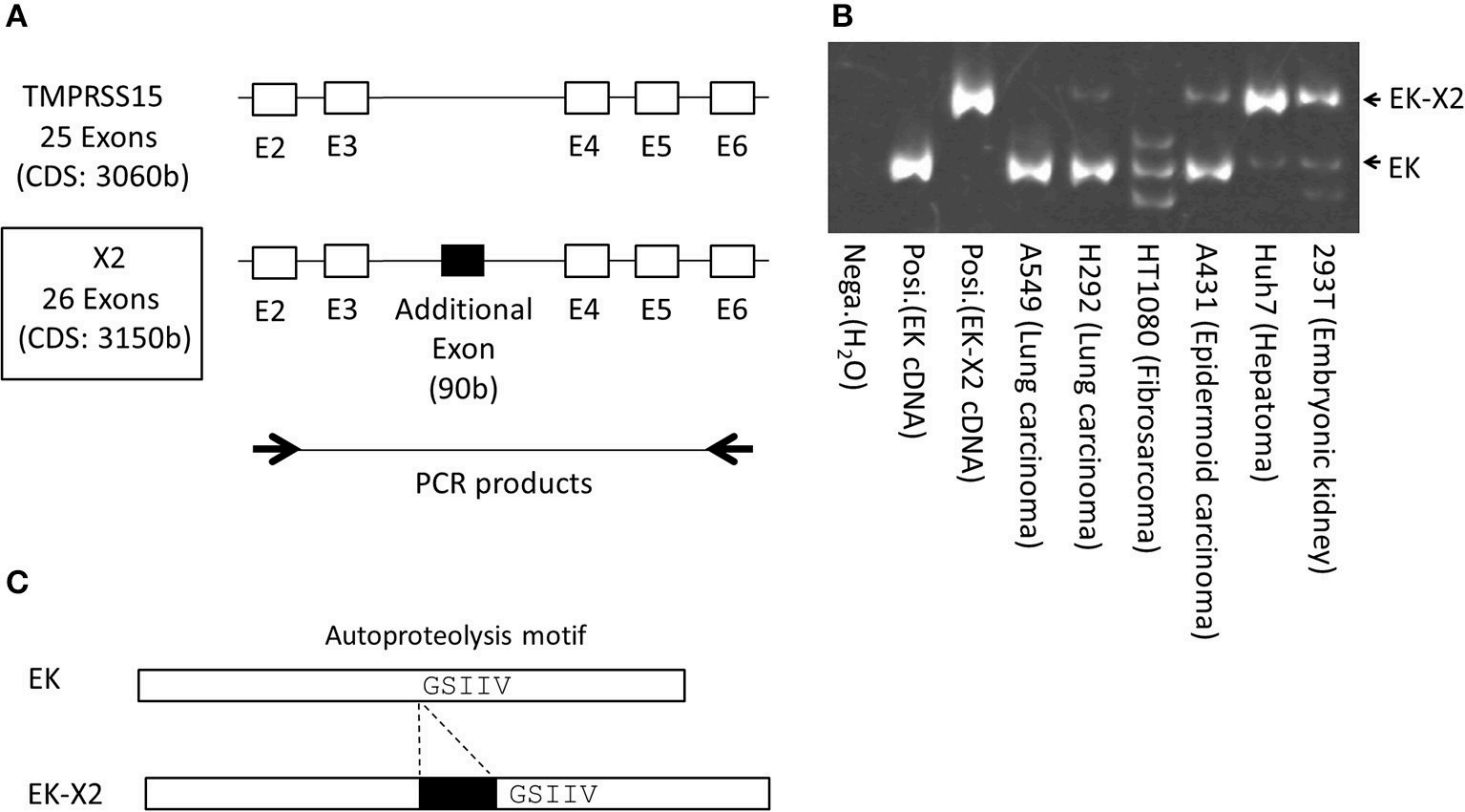
Schematic structures of EK and EK-X2, and their expression in various human cells. **(A)** Exons 2–6 of canonical EK (NM_002772.2) and the additional exon of the EK-X2 isoform (XM_011529657.1) are shown, as in Figure 3. Thick arrows indicate the primer sites in exon 2 and exon 6 of EK used for RT–PCR. **(B)** RT–PCR products after 45 cycles of cDNA amplification (293T, embryonic kidney; Huh7, hepatoma; A431, epidermoid carcinoma; HT1080, fibrosarcoma; H292, lung carcinoma; A549, lung carcinoma), using the primers shown in **(A)**, were separated electrophoretically on a 6% acrylamide gel and stained. Arrows indicate the PCR products of EK (491 bp) and EK-X2 (581 bp). Posi.: 1 × 10^4^ molecules of canonical EK cDNA were used as each positive control. Nega.: H_2_O. **(C)** In the EK-X2 isoform, an additional 90-bp exon is inserted immediately before the autoproteolysis motif (GSIIV) in the SEA domain of canonical EK.

To clarify the role of EK in IAV infection, we cloned the full-length EK cDNAs. The 5′ region of the EK gene is shown in Figure 3. In addition to the canonical EK (TMPRSS15: accession number NM_002772.2), four isoforms (X1–X4) with six transcript variants (accession numbers XM_011529654.1–011529659.1) of EK were predicted using an automated computational analysis of the genomic sequence available in the NCBI GenBank database (https://www.ncbi.nlm.nih.gov/genbank/). The three transcript variants (XM_011529654.1–011529656.1) each encode the same protein isoform (X1) but with different 5′ noncoding regions, and the differences in the coding regions of the X1–X4 isoforms arise from splicing differences between exon 3 and exon 6, including an additional two exons in the canonical EK. We cloned the full-length cDNA encoding the canonical EK from A549 lung-derived cell mRNA (NM_002772.2), and the full-length X2 isoform cDNA from Huh7 liver-derived cells (XM_011529657.1; boxed in Figure 3). Thus, we have designated the canonical cDNA as “EK” and the X2 isoform as “EK-X2.” To further examine the expression profiles of these EK variants in human cell lines, we synthesized specific primers that anneal to exons 2 and 6 of the canonical EK (Figure 4). The expected lengths of the PCR products from EK and EK-X2 were 491 and 581 bp, respectively. The size difference is caused by the insertion of an additional 90-bp exon immediately before the autoproteolysis motif in the SEA (sea urchin sperm protein, enterokinase, and agrin) domain of EK (Figure 4C). Canonical EK was endogenously expressed in A549, H292, HT1080, and A431 cells, whereas the EK-X2 isoform was endogenously expressed in A431, Huh7, and 293T cells. Judging from the extra bands, HT1080 cells may express other isoforms in addition to canonical EK (Figures 3, 4B). These data indicate that canonical EK and its isoforms (at least the X2 isoform) are expressed in many cells other than duodenal cells.

### Exogenous expression of EK or EK-X2 enhances IAV proliferation

**Figure 5:**
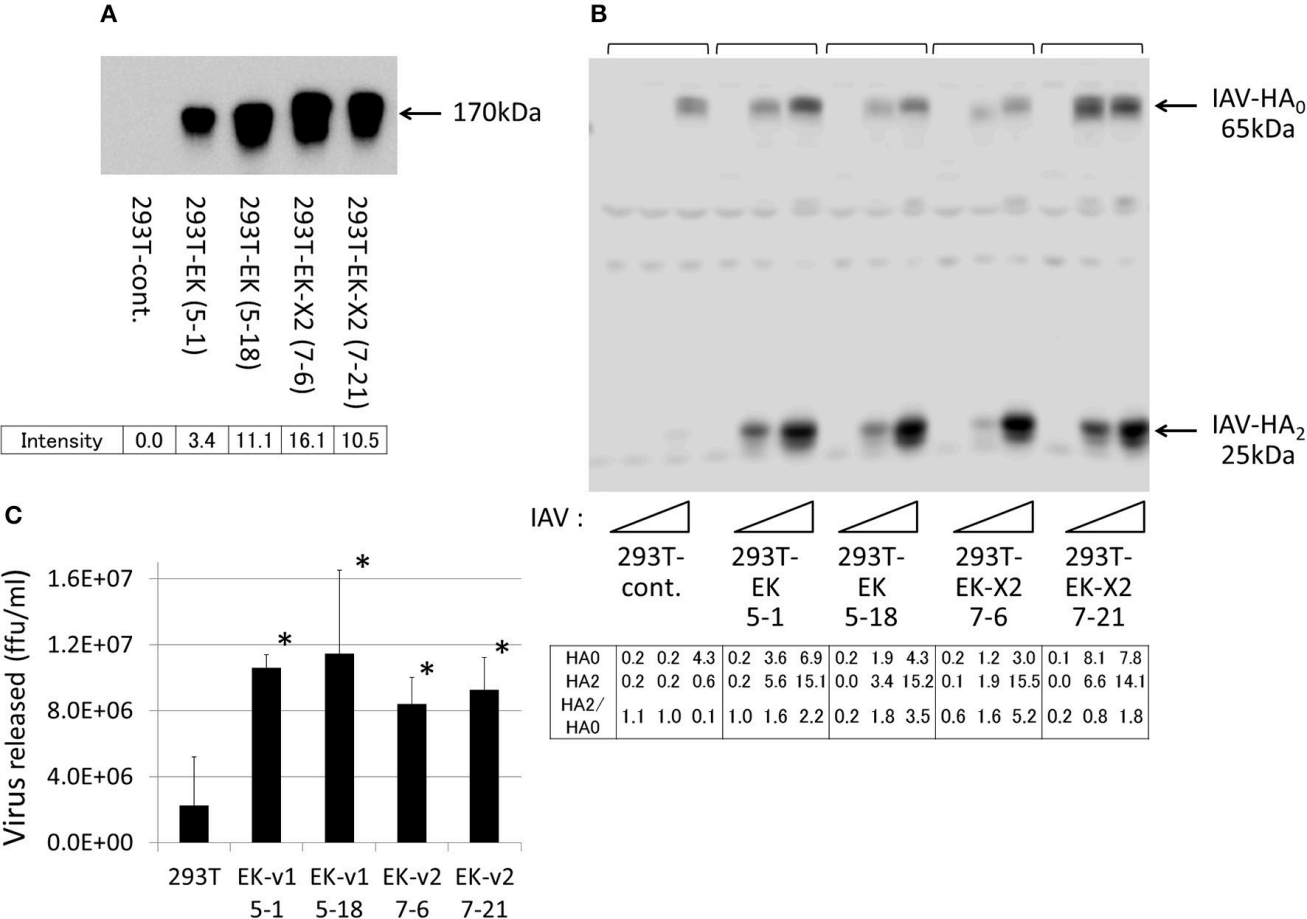
Enhanced proliferation of IAV in 293T cells exogenously expressing EK. **(A)** Two independent 293T clones, stably expressing EK or EK-X2, were isolated by drug selection, as described in section Materials and Methods. The same cell lysate amounts were electrophoresed and then western blotted with an anti-EK antibody. The major EK protein band (170 kDa) and the slightly larger EK-X2 band are indicated by arrows, and the band intensities are shown below. **(B)** 293T cells (1 × 10^5^) in a 24-well plate were inoculated with different amounts of IAV (MOI = 0, 0.001, or 0.01 from the left of 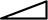). At 48 h post-infection, the same cell lysate amounts were electrophoresed and western blotted with an anti-IAV HA antibody. The 65-kDa precursor IAV HA_0_ and the cleaved 25-kDa C-terminal IAV HA_2_ fragment are indicated by arrows. The HA_0_ and HA_2_ intensities and the HA_2_/HA_0_ ratio are shown below. **(C)** 293T cells (1 × 10^5^) in a 24-well plate were inoculated with IAV (MOI = 0.001). At 48 h post-infection, the viral spread was quantified as the release of infectious particles into the culture supernatants, as measured by a focus forming assay in MDCK cells. Data represent mean ± standard deviation of three experiments, ^*^significantly different at *p* < 0.05.

**Figure 6:**
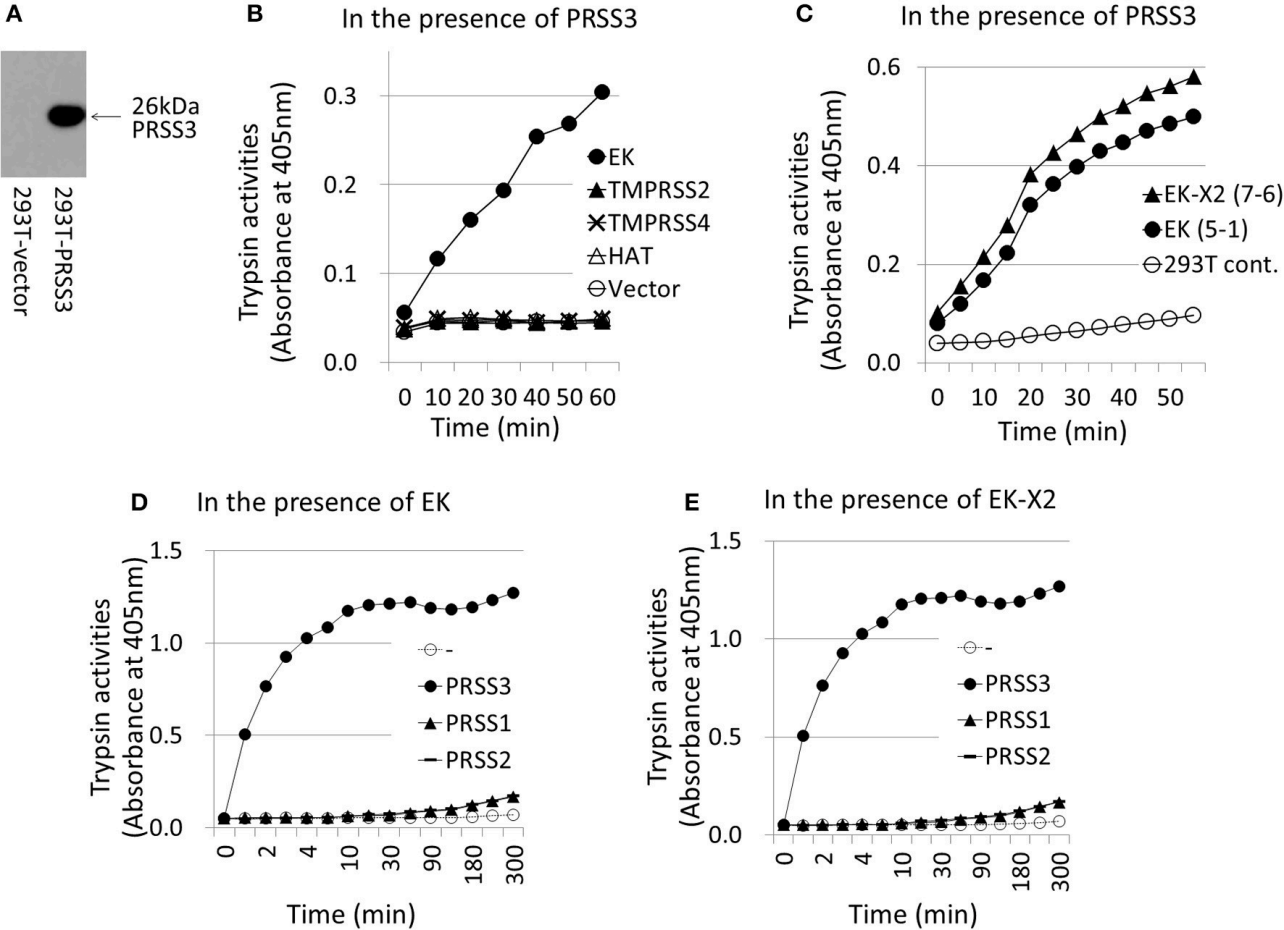
Activation of trypsinogen PRSS3 by TMPRSSs. The activation of trypsinogen PRSS3 by TMPRSSs was measured *in vitro* by incubating PRSS3-expressing cell lysates with a specific substrate and cell lysates expressing the indicated TMPRSSs. **(A)** 293T cells transfected with a PRSS3-expressing plasmid or vector alone were lysed at 48 h post-transfection, electrophoresed, and then western blotted with an anti-PRSS3 antibody. The 26-kDa PRSS3 band is indicated with arrow. **(B)** The PRSS3-expressing 293T cell lysate was mixed with 293T cell lysate transiently expressing one of the indicated TMPRSS proteases (EK, TMPRSS2, TMPRSS4, HAT, or vector only) for the indicated periods. The amount of pNA released from the specific substrate in the mixture was measured at 405 nm as the trypsinogen-activating function (trypsin activity). **(C)** Lysates prepared from 293T cells stably expressing EK (clone #5-1), EK-X2 (clone #7-6), or vector alone (293T-cont.), shown in Figure 5, were incubated with PRSS3-epressing 293T cell lysate, and the trypsinogen-activating function was measured. **(D,E)** 293T cell lysates expressing EK **(D)** or EK-X2 **(E)** were mixed with 293T cell lysates transiently expressing one of the indicated trypsinogens (PRSS1, PRSS2, PRSS3, or vector only) for the indicated periods. The amount of pNA released from the specific substrate in the mixture was measured at 405 nm as the trypsinogen-activating function (trypsin activity).

To examine the effects of the EKs on IAV infection, we expressed EK or EK-X2 exogenously in 293T cells (Figure 5A). We analyzed two independent, stable clones for each construct to avoid clonal deviation. The major EK protein band (170 kDa), and a slightly larger EK-X2 protein band, were detected in the transfected cells using a specific anti-EK antibody. To assess the extent of HA processing quantitatively, we measured the intensities of HA_0_ and HA_2_, and the HA_0_/HA_2_ ratio (Figure 5B). Although the amounts of the 65-kDa HA_0_ precursor and the 25-kDa cleaved C-terminal HA_2_ fragment showed increases, the 25-kDa HA_2_ fragment showed a much more stronger increase (thereby elevating the HA_0_/HA_2_ ratio) after EK or EK-X2 were exogenously expression compared with the 65-kDa HA_0_ precursor, indicating that EK and EK-X2 enhanced not only IAV proliferation, but also HA processing. The EK-mediated proliferation of infective IAV was confirmed by the significant increase of virus release into the media (Figure 5C). We also analyzed the function of EK in activating the PRSS3 trypsinogen *in vitro* (Figure 6). A lysate of 293T cells exogenously expressing a large amount of PRSS3 (Figure 6A) was used as the source of trypsinogen. Although 293T cells endogenously express certain levels of PRSS1 and PRSS3, the lysate exogenously expressing PRSS3 alone (PRSS3 trypsinogen lysate) showed no catalytic activity for a pNA-attached trypsin substrate. Consequently, the authenticity of the substrate was confirmed by the observation that free pNA was markedly released when the PRSS3 lysate was incubated with a purified EK (GenScript, #Z01003). Each 293T cell lysate exogenously expressing EK, TMPRSS2, TMPRSS4, HAT proteases, or vector alone, was mixed with the PRSS3 lysate and the specific trypsin substrate to measure PRSS3 activation by the transmembrane proteases (Figure 6B). The EK lysate was able to activate PRSS3 in a time-dependent manner. In contrast, TMPRSS2, TMPRSS4, and HAT were all unable to activate PRSS3, which indicates that only EK can cleave and activate the PRSS3 trypsinogen. A trypsinogen-activating function was also detected in the 293T cell lysates stably expressing EK or EK-X2 (Figure 6C). EK- and EK-X2-mediated activation of PRSS1 and PRSS2 produced marginal results, unlike that with PRSS3 (Figures 6D,E). These findings indicate that PRSS3 is a major activator of EK and EK-X2 *in vitro*.

**Figure 7:**
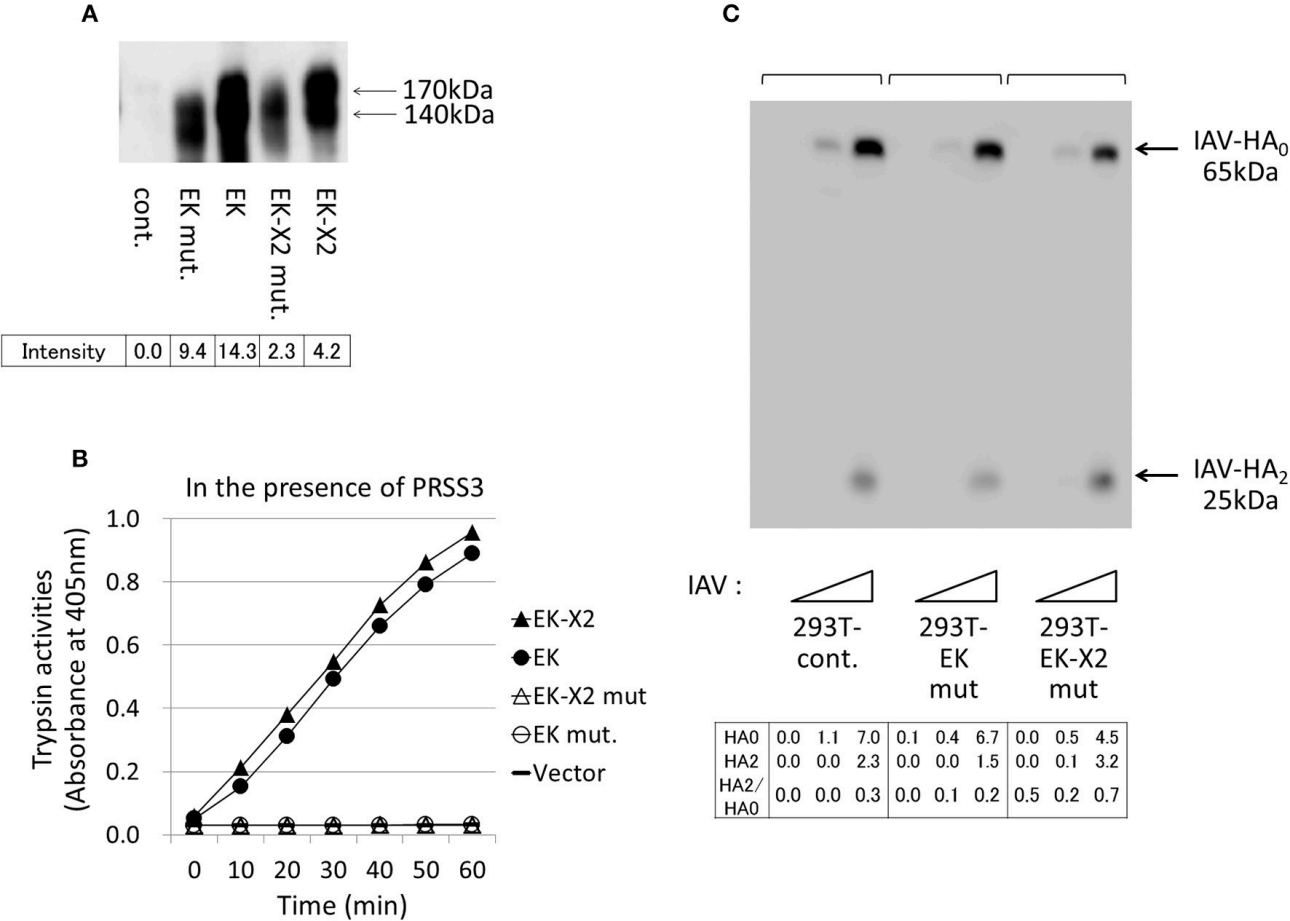
Protease-deficient mutant EK or EK-X2 has almost no effect on IAV infection. **(A)** The protease-deficient mutants in which Ala replaced EK Ser^971^ or EK-X2 Ser^1001^ in the catalytic triad (H–D–S), as well as wild type EK and EK-X2, were transiently expressed in 293T cells, and the cell lysates were analyzed with western blotting using an anti-EK antibody. The two major protein bands of EK (170 and 140 kDa) and the EK mutant and the slightly larger bands of EK-X2 and its mutant are indicated with arrows, and the band intensities are shown below. **(B)** The 293T cell lysates transiently expressing wild type EK, EK-X2, or the protease-deficient mutants were mixed with the PRSS3-expressing 293T cell lysate. The amounts of pNA released from the substrate were measured at 405 nm as the trypsinogen-activating function. **(C)** 293T and mutant EK- or EK-X2-expressing cells (1 × 10^5^ each) were plated in a 24-well plate and inoculated with different amounts of IAV (MOI = 0, 0.001, 0.01 from the left of 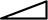). At 48 h after infection, the same amounts of cell lysates were subjected to electrophoresis and western blotting with an anti-IAV HA antibody. The 65-kDa precursor IAV HA_0_ and the cleaved 25-kDa C-terminal IAV HA_2_ fragment are indicated by arrows. The HA_0_ and HA_2_ intensities and the HA_2_/HA_0_ ratio are shown below.

The His^825^-Asp^876^-Ser^971^ residues in canonical EK form the evolutionarily-conserved H–D–S catalytic triad present in many serine proteases (Choi et al., 2009). We constructed protease-deficient mutants by replacing Ser^971^ of EK and Ser^1001^ of EK-X2 with Ala. Upon electrophoresis, the mutant proteins were a little smaller than the wild-type proteins (Figure 7A). Although the expression levels of the mutant proteins were more than halve of those of the parental proteins in 293T cells, neither of the mutant EKs displayed enzymatic activity (Figure 7B) or any enhancing effect on HA processing or IAV proliferation (Figure 7C) in the 293T cells compared with those of the control. This indicates that the enhancing effects of EK and EK-X2 on IAV infection depend on their PRSS3-activating functions.

**Figure 8:**
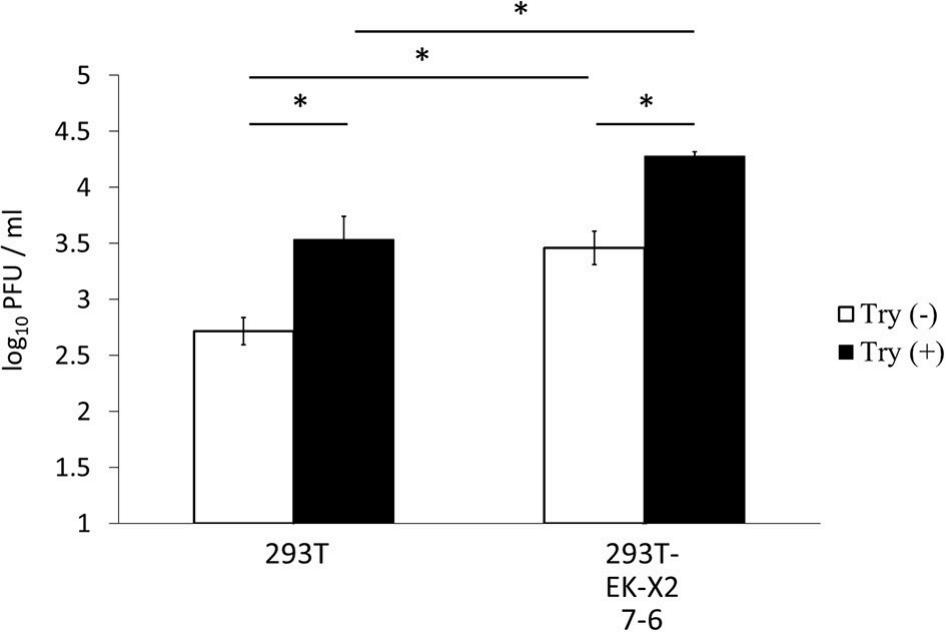
Effects of EK-X2 on infection by another IAV strain, A/Aichi/2/68 (H3N2). The effects of EK-X2 expression on infection by another IAV strain, A/Aichi/2/68 (H3N2), were examined. Levels of IAV proliferation in 239T and EK-X2-expressing 293T cells (#7-6) 48 h after infection with the virus (MOI = 0.1), with or without 5 μg/ml trypsin, were measured with a plaque assay in MDCK cells. Data represent mean ± standard deviation of three experiments. ^*^Significantly different (*p* < 0.05).

Because the trypsin requirement differs among viral strains, we investigated the effects of EK expression on infection with another IAV strain [A/Aichi/2/68 (H3N2)]. The A/Aichi/2/68 (H3N2) strain requires more trypsin to enhance its proliferation in host cells than the A/WSN/1933(H1N1) strain (Goto and Kawaoka, 1998). The level of IAV proliferation 48 h after infection at a multiplicity of infection (MOI) of 0.1, as measured by a plaque assay in MDCK cells, was significantly higher in the EK-X2-expressing 293T cells (clone #7-6) than in the 293T cells (Figure 8). Treatment of the IAV strain with trypsin significantly enhanced viral proliferation over 48 h in both 293T and EK-X2-expressing 293T cells (#7-6). The marked increase in IAV proliferation in the 293T cells without trypsin treatment indicates that EK-X2 expression in these cells was able to enhance the proliferative ability of the Aichi strain (in addition to the WSN strain), although the additional enhancing effect of trypsin treatment in the EK-X2-expressing cells suggests that the EK-X2-dependent viral proliferation in the cells was partial and incomplete.

### EK and EK-X2 trigger HA processing by activating the PRSS3 trypsinogen

**Figure 9:**
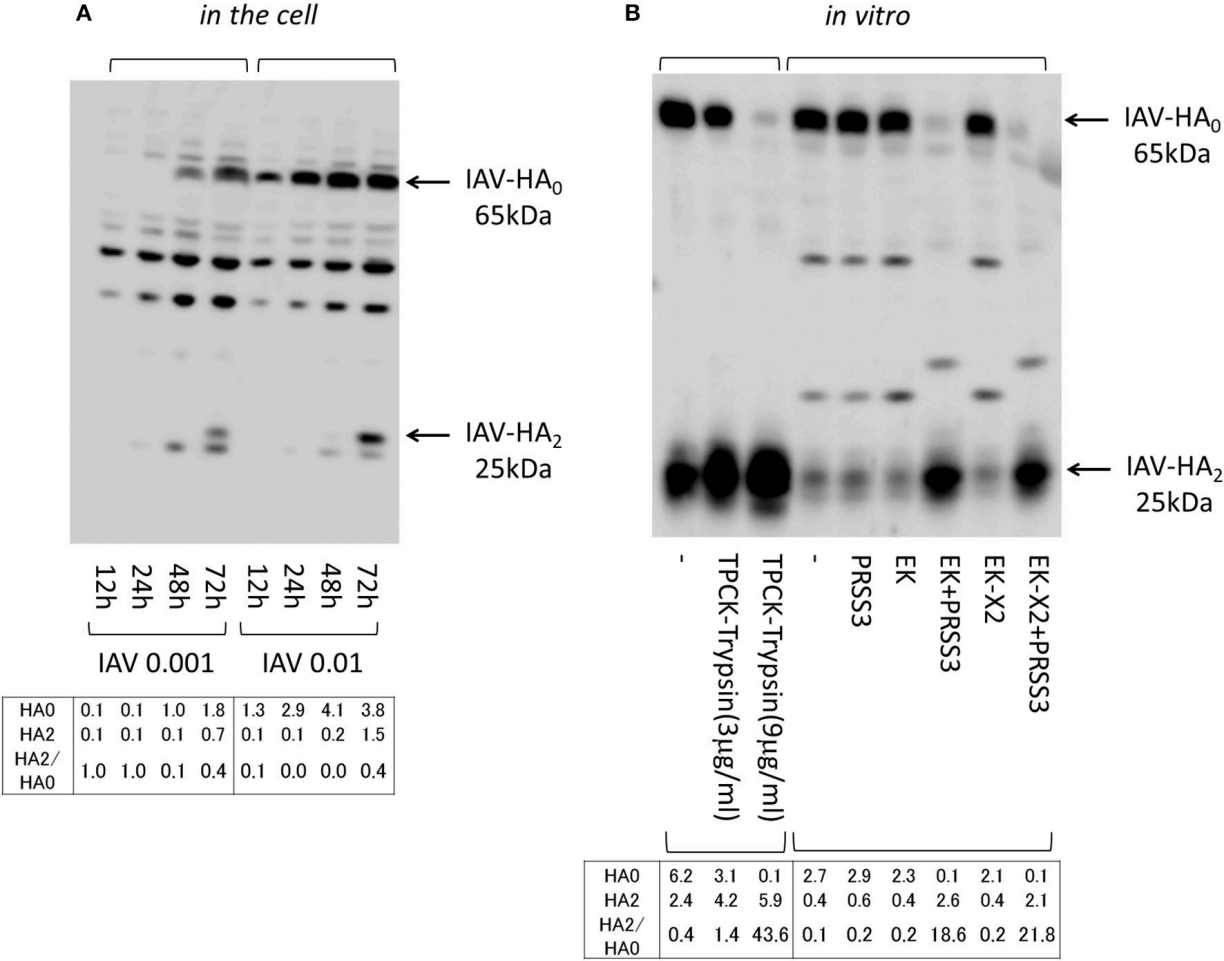
Processing IAV HA. **(A)** 293T cells (1 × 10^5^) plated in a 24-well plate were inoculated with IAV (MOI = 0.001, 0.01). Cell lysates were prepared after the indicated periods and subjected to electrophoresis and western blotting with an anti-IAV HA antibody. **(B)** IAV HA_0_ was prepared from IAV-infected U937 lymphoma cells (MOI = 0.1), which permit IAV proliferation, and was used in processing experiments *in vitro*. IAV HA_0_ lysates were incubated for 30 min at room temperature with TPCK-treated trypsin (0, 3, or 9 μg/ml) or with the indicated lysates expressing PRSS3, EK, EK-X2, or vector only (–), and then subjected to western blotting with an anti-IAV HA antibody. The 65-kDa precursor IAV HA_0_ and the cleaved 25-kDa C-terminal IAV HA_2_ fragment are indicated by arrows. The HA_0_ and HA_2_ intensities and the HA_2_/HA_0_ ratio are shown below.

We next examined the effects of the EKs on HA processing, when inactive HA_0_ is converted to the HA_1_ and HA_2_ active forms via cleavage at a specific arginine residue, both *in vitro* and in the infected cells. To access the effect of the exogenously expressed EKs on HA processing, we first analyzed the dynamics of the HA molecules after the 293T cells were infected with different amounts of IAV (MOI = 0.001, 0.01). Cell proteins were western blotted with an anti-HA antibody that detects the 65-kDa HA_0_ and the C-terminal 25-kDa HA_2_ fragment (Figure 9A). The 65-kDa HA_0_ protein was detected in the cell lysates 12 h after IAV infection (MOI = 0.01), and the processed 25-kDa HA_2_ fragment was observed 72 h after the cells were infected with IAV. HA processing proceeded in a time- and dose-dependent manner in the 293T cells, and was enhanced by the presence of EK and EK-X2 alike, as has already been shown in Figure 5. We next examined HA processing *in vitro*, by preparing IAV from U937 lymphoma cells because the amount of IAV-HA_0_ was higher than in the 293T cells where marginal HA_0_ processing occurs, even at 48 h post-infection (Figure 1). The IAV-HA_0_-rich cell lysates were incubated with TPCK-treated trypsin or lysates containing EK (Figure 9B). The trypsinogen PRSS3, EK, or EK-X2 alone had no effect on HA_0_, but HA_0_ disappeared and HA_2_ appeared correspondingly after treating PRSS3 with EK or EK-X2, as also occurred after treatment with TPCK-treated trypsin. This indicates that both EK and EK-X2 cleaved and activated trypsinogen to process HA_0_ into HA_1_ and HA_2_ *in vitro* and in the cells.

### Endogenous EK in A549 lung-derived cells plays a role in IAV infection

To evaluate the role of endogenous EK in IAV infections, we used A549 lung-derived cells because the lungs are a primary target of IAV, and because these cells express a certain level of EK, but do not express TMPRSS2, TMPRSS4, or HAT (Figure 2A). However, the HA expression level was relatively low in the A549 cells, compared with the 293T and Huh7 cells. Therefore, to clarify the effects of the EK knockdown, we first established an A549 cell line that permitted good proliferation IAV by knocking down the critical signaling molecules that trigger interferon production by sensing IAV infection. Among these signaling molecules (data not shown), the RIG-I knockdown was the most effective at enhancing IAV proliferation and HA expression.

**Figure 10:**
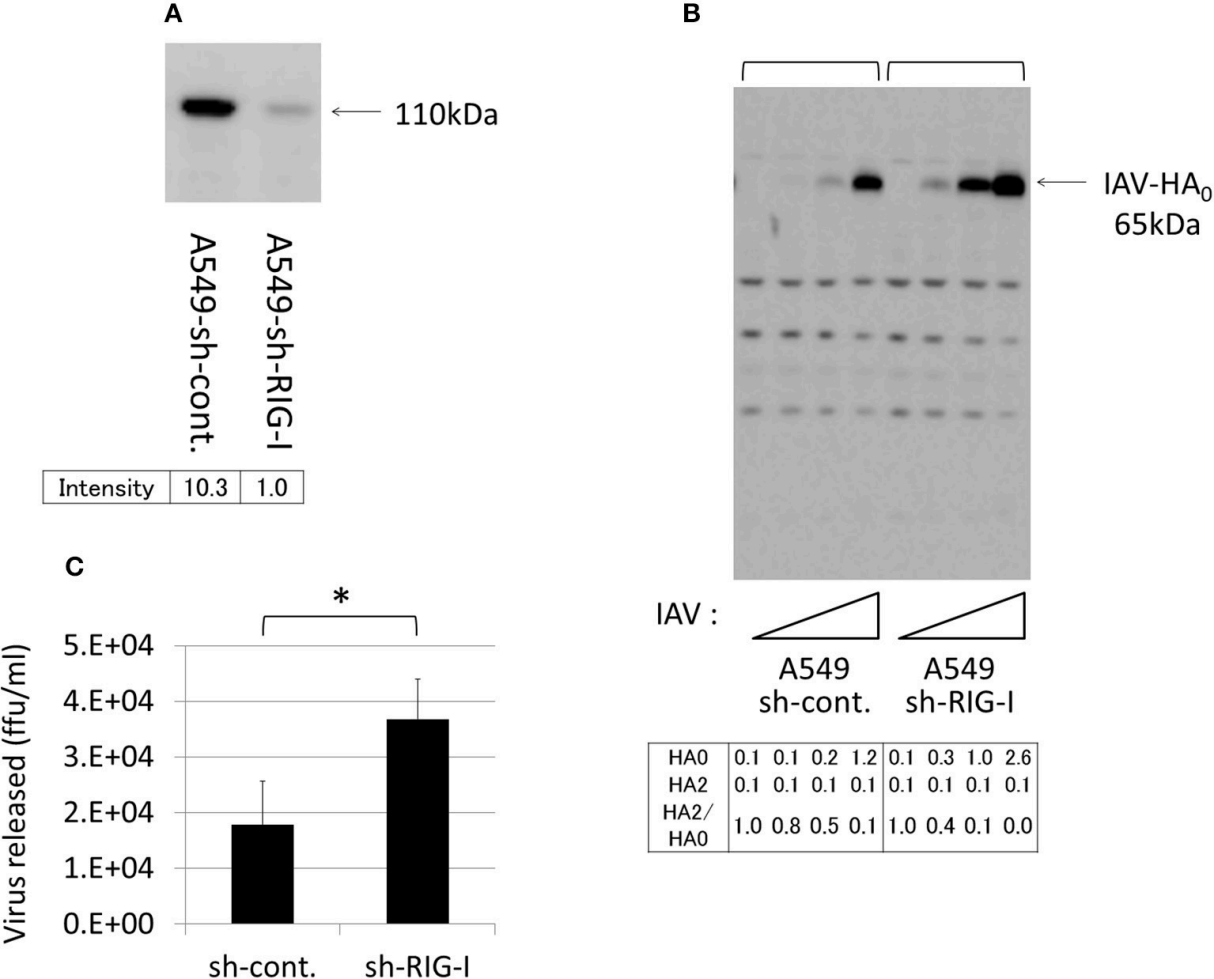
Knockdown of RIG-I enhanced IAV proliferation in A549 cells. **(A)** A549 cells stably expressing the control shRNA (sh-cont.) or the shRNA confirmed as targeting RIG-I mRNA (sh-RIG-I) were generated by infection with an shRNA-expressing recombinant lentivirus and drug selection, as described in the section Materials and Methods. To verify the shRNA targeting RIG-I, the cell lysates were subjected to western blotting with an anti-RIG-I antibody. The 110-kDa RIG-I band is indicated with an arrow, and the band intensity is shown below. **(B)** A549 cells were infected with IAV (MOI = 0, 0.001, 0.01, 0.1 from the left of 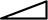) for 48 h, and the proliferation of IAV was analyzed with western blotting using an anti-HA antibody. The 65-kDa precursor IAV HA_0_ is indicated with arrows. Intensities of HA_0_ and HA_2_, and the HA_2_/HA_0_ ratio were shown below. **(C)** The indicated A549 cells (1 × 10^5^) in a 24-well plate were inoculated with IAV (MOI = 0.001). At 48 h post-infection, the viral spread was quantified as the release of infectious particles into the culture supernatants, as measured by a focus forming assay in MDCK cells. Data represent mean ± standard deviation of three experiments, ^*^Significantly different at *p* < 0.05.

**Figure 11:**
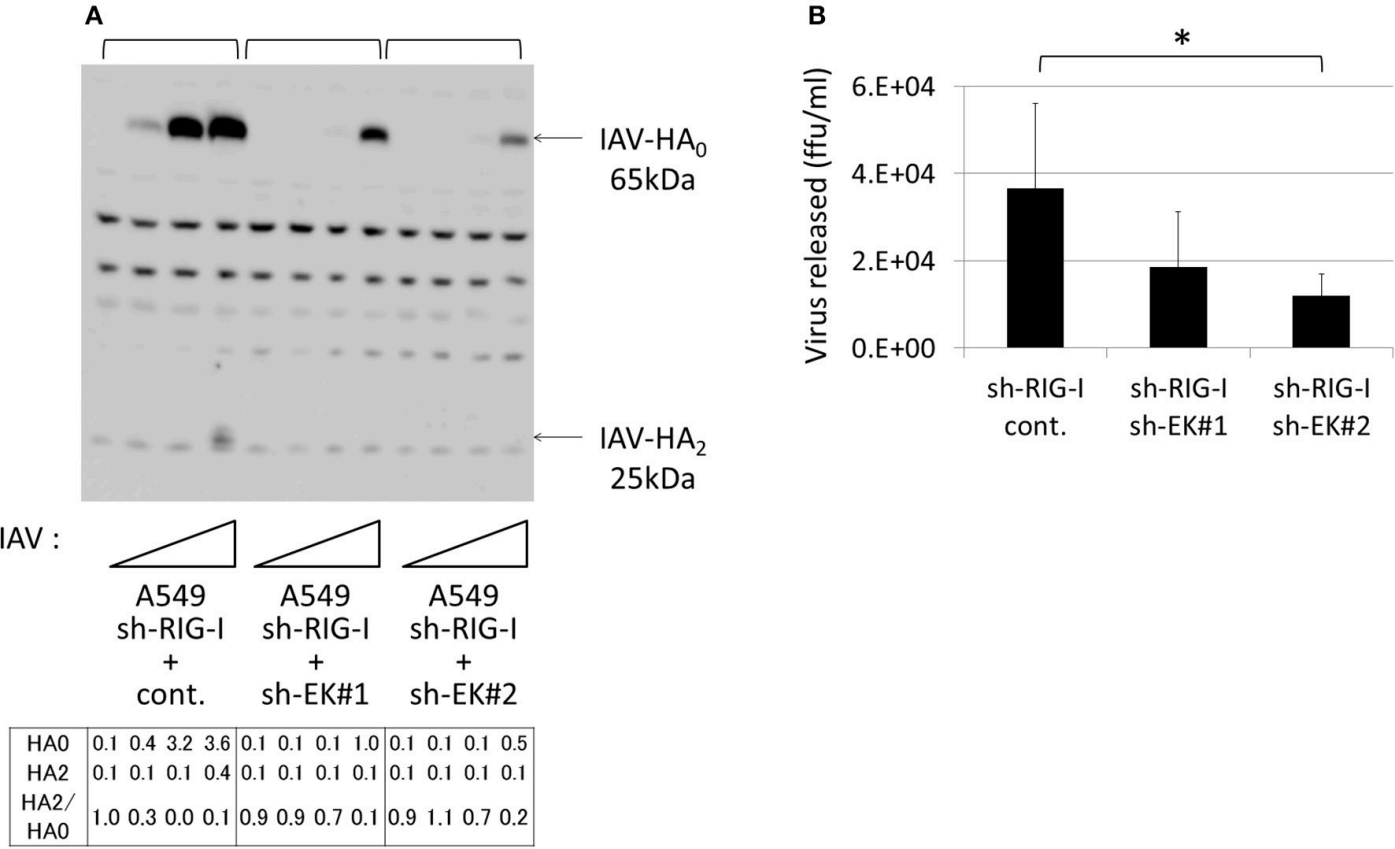
Knockdown of endogenous EK in the A549 cells expressing sh-RIG-I reduced IAV proliferation. **(A)** sh-RIG-I-expressing A549 cells also expressing shRNAs targeting different sites in EK (sh-EK#1, sh-EK#2, or sh-cont.) were generated by infecting the cells with a recombinant lentivirus expressing an shRNA and a blasticidin-resistant gene. The drug-resistant cells were pooled and infected with IAV (MOI = 0, 0.001, 0.01, 0.1 from the left of 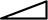). At 48 h post-infection, the same cell lysate amounts were electrophoresed and western blotted with an anti-IAV HA antibody. The 65-kDa precursor IAV HA_0_ and the cleaved 25-kDa C-terminal IAV HA_2_ fragment are indicated by arrows. The HA_0_ and HA_2_ intensities and the HA_2_/HA_0_ ratio are shown below. **(B)** A549 cells (1 × 10^5^) in a 24-well plate were inoculated with IAV (MOI = 0.001). At 48 h post-infection, the viral spread was quantified as the release of infectious particles into the culture supernatants, as measured by a focus forming assay in MDCK cells. Data represent mean ± standard deviation of three experiments. ^*^Significantly different at *p* < 0.05.

Thus, to establish A549 cells capable of stably expressing shRNA directed against RIG-I (A549–sh-RIG-I), we infected A549 cells with a recombinant lentivirus expressing the verified sh-RIG-I and a puromycin-resistance gene. The drug-resistant cells were pooled to avoid clonal deviation while establishing the stable cell lines. We noted that the RIG-I protein levels reduced significantly when shRNA was expressed in the cells (Figure 10A). Although HA processing was not observed clearly, the amount of HA_0_ and the release of infective viruses into the culture media were enhanced in the A549 cells by knocking down the endogenous RIG-I with shRNA (Figures 10B,C). We further synthesized two shRNAs (sh-EK#1 and sh-EK#2) targeting different sites in the EK mRNA to knockdown endogenous EK in the A549 cells with a recombinant lentivirus vector. To avoid clonal deviation in the stable cell lines we established, we pooled the drug-resistant cells expressing the verified EK-directed shRNAs in the A549–sh-RIG-I cells. The sh-EK#2–mediated EK knockdown was more effective at reducing the amount of HA_0_ and the spread of infective virus than that of shEK#1 (Figure 11). These results indicate that endogenous EK plays a role in enhancing IAV proliferation in A549 cells.

## Discussion

**Figure 12:**
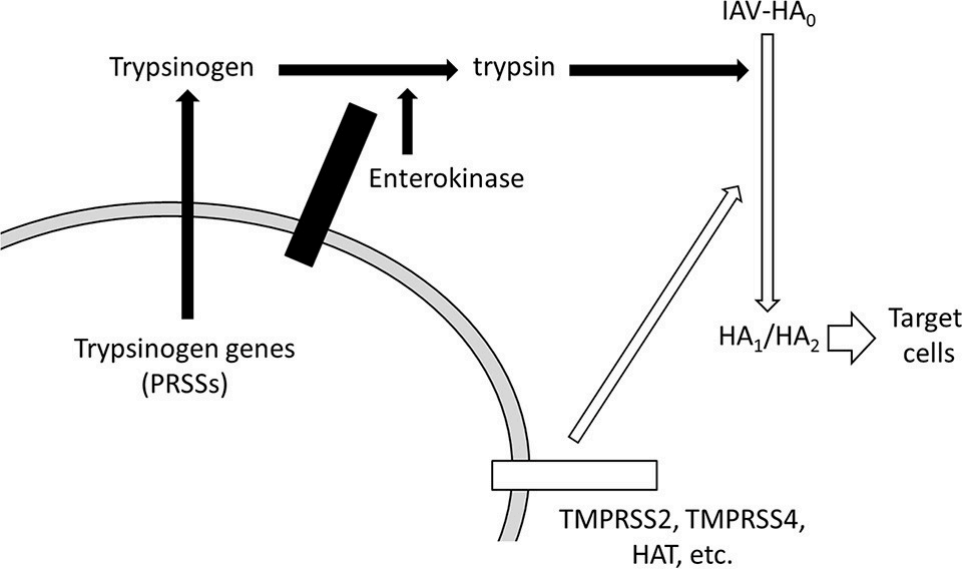
Schematic representation of HA processing by EK and trypsinogen. The novel pathway that enhances IAV infection via EK is shown with solid arrows. EK activates trypsinogen PRSSs to process the precursor HA_0_ into the active HA_1_ and HA_2_ conformations. The known transmembrane serine protease (TMPRSS) pathway, involving TMPRSS2, TMPRSS4, and HAT, which directly activates HA processing, is shown with open arrows.

We have shown that at least two EK isoforms are expressed in human cell lines, including in A549 lung-derived cells. The enhancing effects of exogenously expressed canonical EK or EK-X2 on IAV infection depended on their protease activities, and correlated with HA processing and trypsinogen activation. In addition to the HA-processing pathway via the other TMPRSSs reported to date, we propose a novel pathway that enhances IAV infection via EK, which activates trypsinogen to process the HA_0_ precursor into mature HA_1_ and HA_2_ fragments, as shown schematically in Figure 12.

As shown in Figure 2, lung-derived A549 cells predominantly express EK among the TMPRSSs. This is one explanation for why knocking down endogenous EK in A549 cells resulted in reduced IAV proliferation (Figure 11). In contrast, the EK knockdown in 293T cells had almost no effect on IAV proliferation (data not shown). This is understandable because 293T cells express both TMPRSS2 and EK, and TMPRSS2 can activate HA even in the absence of EK. Among the TMPRSSs, TMPRSS2 (Böttcher et al., 2006; Chaipan et al., 2009; Bertram et al., 2010; Hatesuer et al., 2013; Sakai et al., 2014; Kühn et al., 2016), TMPRSS4 (Chaipan et al., 2009; Bertram et al., 2010; Kühn et al., 2016), HAT (TMPRSS11D) (Böttcher et al., 2006), matriptase (ST14) (Hamilton et al., 2012), DESC1 (TMPRSS11E) (Zmora et al., 2014), and TMPRSS13 (MSPL) (Okumura et al., 2010; Zmora et al., 2014) have all been reported to cleave and activate IAV HA. Additionally, TMPRSS2 has been shown to be a major target of many IAV strains including H1N1 *in vivo* by using knockout mice (Kim et al., 2006; Hatesuer et al., 2013; Sakai et al., 2014), whereas, TMPRSS4 was a target of another IAV strain (H3N1) *in vivo* (Kühn et al., 2016). As for the WSN strain, it uses plasmin to cleave HA_0_ with the help of neuraminidase (NA) (Goto and Kawaoka, 1998). These findings show that host proteases play an important role in determining the cell or tissue tropism of an IAV strain. However, the host protease that is used by IAV depends not only on the expression profiles of the relevant proteases in the targeted cell, but other factors that also regulate protease functions, such as EK-related activation of PRSS3. IAV itself affects the selection process by inducing various cytokines such as interferons, tumor necrosis factor α, and interleukin 1β (IL-1β), or by inhibiting various cellular responses in order to replicate in the cell. For example, IAV-induced IL-1β stimulates the expression of PRSSs, including PRSS3, in several cell types (Indalao et al., 2017), and elevated PRSS3 protein is activated by EKs to induce HA processing. Therefore, EKs may play a role in IAV pathogenicity and cell tropism via PRSS3.

To effectively prevent or cure IAV infection, therapeutic interventions targeting several molecules simultaneously are worth considering. Developing inhibitors against trypsin-like proteases, such as TMPRSS2 and EK, is warranted because targeting viral proteins, including HA and NA, could stimulate the emergence of resistant mutant viruses, given the highly variable nature of IAV. The characteristic structure of EK, whose catalytic domains are exposed on the cell surface, facilitates the development of inhibitors that have minimal adverse effects. The strict substrate specificity of EK for the DDDDK amino acid sequence makes it particularly suitable for circumventing possible adverse effects. The combined use of EK inhibitors with other reagents may exert the maximal damaging effect on IAV during its infection.

Although no functional differences between EK and EK-X2 were apparent in this study, the insertion of an additional 90-bp exon (encoding 30 amino acids) just before the GSIIV autoproteolysis motif might modify the structure and function of the 118 amino acids of the SEA domain in canonical EK (Figure 4C). As its name implies, the canonical SEA domain was first detected in sea urchin sperm protein, and in EK and agrin proteins also, but its function is not well understood, except that it is known to be involved in the autoproteolysis that causes subsequent functional domains to be shed or the protein to be degraded, and it also has an effect on the carbohydrate chains nearby it (List et al., 2006). Recent advanced search programs have shown that many molecules contain the SEA domain, including some mucins, glycans, phosphatases, and cadherins (Pei and Grishin, 2017). Because the 90-bp exon of EK-X2 is inserted immediately before the autoproteolysis motif in EK, it may affect some EK functions as yet unknown or contribute a new function for the SEA domain. It is intriguing that many cell lines express canonical EK, but some express EK-X2, and possibly other variants (Figure 4B). The Human Protein Atlas project provides information on the overall expression levels of EK without differentiating its variants (The Human Protein Atlas, 2017). EK expression at the RNA level in A549 cells is marginal, compared with that in the brain cancer cell lines U-87 MG and U-138 MG. The very high expression level of EK in brain cell lines requires further examination because EK might be involved in IAV-induced encephalopathy or abnormal psychiatric behaviors. The physiological roles of EK, especially in the lung and brain, after IAV infection *in vivo* deserve future research.

## Author contributions

HH: conceived and designed the experiments; HH, ET, and KS: performed the experiments; HH, YK, MI, HK, MY, HN, KN, TM: analyzed the data; HH, YK, and TM: wrote the paper.

### Conflict of interest statement

The authors declare that the research was conducted in the absence of any commercial or financial relationships that could be construed as a potential conflict of interest.
